# Molecular characterization and classification of *Trypanosoma* spp*.* Venezuelan isolates based on microsatellite markers and kinetoplast maxicircle genes

**DOI:** 10.1186/s13071-015-1129-2

**Published:** 2015-10-15

**Authors:** E. Sánchez, T. Perrone, G. Recchimuzzi, I. Cardozo, N. Biteau, PM Aso, A. Mijares, T. Baltz, D. Berthier, L. Balzano-Nogueira, MI Gonzatti

**Affiliations:** Laboratorio de Fisiología de Parásitos. Centro de Biofísica y Bioquímica, Instituto Venezolano de Investigaciones Científicas, Caracas, Venezuela; Grupo de Bioquímica e Inmunología de Hemoparásitos. Departamento de Biología Celular, Universidad Simón Bolívar, Caracas, 1080 Venezuela; Laboratoire de Microbiologie Fondamentale et Pathogénicité, Université Bordeaux. UMR-CNRS 5234, 146, Rue Léo Saignat, 33076 Bordeaux, Cedex France; CIRAD, UMR InterTryp, F-34398 Montpellier, France; Laboratorio de Biometría y Estadística, Área de Agricultura y Soberanía Alimentaria, Instituto de Estudios Avanzados, Caracas, 1015A Venezuela

**Keywords:** *Trypanosoma equiperdum*, *Trypanosoma evansi*, Maxicircle, Microsatellite genotyping, Coinertia analysis

## Abstract

**Background:**

Livestock trypanosomoses, caused by three species of the Trypanozoon subgenus, *Trypanosoma brucei brucei*, *T. evansi* and *T. equiperdum* is widely distributed throughout the world and constitutes an important limitation for the production of animal protein. *T. evansi* and *T. equiperdum* are morphologically indistinguishable parasites that evolved from a common ancestor but acquired important biological differences, including host range, mode of transmission, distribution, clinical symptoms and pathogenicity. At a molecular level, *T. evansi* is characterized by the complete loss of the maxicircles of the kinetoplastic DNA*,* while *T. equiperdum* has retained maxicircle fragments similar to those present in *T. brucei. T. evansi* causes the disease known as Surra, Derrengadera or "mal de cadeiras", while *T. equiperdum* is the etiological agent of dourine or "mal du coit", characterized by venereal transmission and white patches in the genitalia.

**Methods:**

Nine Venezuelan *Trypanosoma spp*. isolates, from horse, donkey or capybara were genotyped and classified using microsatellite analyses and maxicircle genes. The variables from the microsatellite data and the Procyclin PE repeats matrices were combined using the Hill-Smith method and compared to a group of *T. evansi*, *T. equiperdum* and *T. brucei* reference strains from South America, Asia and Africa using Coinertia analysis. Four maxicircle genes (cytb, cox1, a6 and nd8) were amplified by PCRfrom TeAp-N/D1 and TeGu-N/D1, the two Venezuelan isolates that grouped with the T. *equiperdum* STIB841/OVI strain. These maxicircle sequences were analyzed by nucleotide BLAST and aligned toorthologous genes from the Trypanozoon subgenus by MUSCLE tools. Phylogenetic trees were constructed using Maximum Parsimony (MP) and Maximum Likelihood (ML) with the MEGA5.1® software.

**Results:**

We characterized microsatellite markers and Procyclin PE repeats of nine Venezuelan *Trypanosoma* spp. isolates with various degrees of virulence in a mouse model, and compared them to a panel of *T. evansi* and *T. equiperdum* reference strains. Coinertia analysis of the combined repeats and previously reported *T. brucei brucei* microsatellite genotypes revealed three distinct groups. Seven of the Venezuelan isolates grouped with globally distributed *T. evansi* strains, while TeAp-N/D1 and TeGu-N/D1 strains clustered in a separate group with the *T. equiperdum* STIB841/OVI strain isolated in South Africa. A third group included *T. brucei brucei*, two strains previously classified as *T. evansi* (GX and TC) and one as *T. equiperdum* (BoTat-1.1). Four maxicircle genes, Cytochrome b, Cythocrome Oxidase subunit 1, ATP synthase subunit 6 and NADH dehydrogenase subunit 8, were identified in the two Venezuelan strains clustering with the *T. equiperdum* STIB841/OVI strain. Phylogenetic analysis of the *cox1* gene sequences further separated these two Venezuelan *T. equiperdum* strains: TeAp-N/D1 grouped with *T. equiperdum* strain STIB818 and *T. brucei brucei*, and TeGu-N/D1 with the *T. equiperdum* STIB841/OVI strain.

**Conclusion:**

Based on the Coinertia analysis and maxicircle gene sequence phylogeny, TeAp-N/D1 and TeGu-N/D1 constitute the first confirmed *T. equiperdum* strains described from Latin America.

**Electronic supplementary material:**

The online version of this article (doi:10.1186/s13071-015-1129-2) contains supplementary material, which is available to authorized users.

## Background

Trypanosomes are unicellular parasites that cause important diseases in humans and animals. They comprise a wide group of parasites of vertebrates usually transmitted by haematophagous arthropods [1]. Livestock trypanosomoses caused by *Trypanosoma brucei brucei, Trypanosoma equiperdum* and *Trypanosoma evansi,* all belonging to the Trypanozoon subgenus, has a significant socio-economic impact and limits animal protein productivity throughout the world [2–4]. *T. evansi*, the first pathogenic trypanosome discovered, was found in the blood of horses and camels suffering an endemic disease known as Surra, derrengadera, murrina or “mal de cadeiras” [5]. *T. evansi* was introduced to America in the XV century through the importation of horses from Africa by Spanish conquerors [5–7]. *T. evansi* is mechanically transmitted and it is now widely distributed throughout tropical and subtropical regions of Northern Africa, Southeast Asia, as well as Central and South America, where livestock and native wildlife are severely affected [2, 5, 8–10]. In Europe, the importation of dromedary camels from the Canary Islands was associated with *T. evansi* outbreaks in France and Spain [11, 12] and chronic *T. evansi* infection and death was reported in a dog that returned to Germany after international travel [13].

*T. evansi* is morphologically indistinguishable from the two other pathogenic species, *T. brucei* and *T. equiperdum* [5, 14–17]. *T. equiperdum* is the causative agent of dourine, a distinctive disease that only affects *Equidae* and is transmitted mainly via coitus [16, 18–22]. Because *T. evansi*, *T. equiperdum* and *T. brucei* species cannot be distinguished by sequences of their ribosomal RNA (rRNA) genes [1, 23–27], other probes, including isoenzymes and peptidase profiles [28–30], restriction fragment length polymorphisms [31], kinetoplast sequences [32, 33] and microsatellites markers [34] have been used to characterize and distinguish these trypanosome species. Simple sequence repeat (SSR) microsatellites are DNA loci with tandemly repeated short sequence motifs, whose copy number is hypervariable at each locus [35, 36]. The mutation rate of SSR microsatellites, and thus their variability, is higher than that observed for isoenzyme or RFLP markers [37–39], making them highly useful for studying the relationships between closely related species or within populations of the same species [40, 41].

All Kinetoplastids, including the species within the Trypanozoon subgenus, contain kinetoplasts, a concatenated network of complex mitochondrial DNA comprising 5.000-10.000 minicircles of about 1000 bp [42], *T. brucei* minicircles sequences are highly heterogeneous [43] while the minicircles from *T. evansi* strains from Africa, Asia and South America, show extensive sequence conservation [44–47]. With the exception of *Trypanosoma evansi,* all species within the Trypanosoma genus contain 50-100 complete or partial maxicircles varying in size from 20 Kbp for *T. brucei* ssp*.* to 40 kbp for *C. fasciculata* [48]. The maxicircles encode mitochondrial genes necessary for development and differentiation in the insect vector [48–51].

We previously showed that Venezuelan *Trypanosoma* spp. isolates separated into two groups, according to their RAPD profiles [52]. Seven of the nine Venezuelan isolates clustered together, while two highly virulent horse isolates, TeAp-N/D1 and TeGu-N/D1, appeared to be genetically distinct [17, 52, 53]. By analyzing microsatellite loci and PE repeats, we found that the seven less virulent isolates clustered with *T. evansi* reference strains, while the more virulent TeAp-N/D1 and TeGu-N/D1 isolates closely matched a *T. equiperdum* reference strain, leading us to look for the presence of maxicircle genes.

## Methods

### *Trypanosoma* spp*. field isolates and DNA extraction*

Nine Venezuelan *Trypanosoma* spp*.* isolates were obtained from horse, donkey or capybara blood samples, as previously described [52]: TeAp-Cedral05, TeAp-Cedral12, TeAp-ElFrío01, TeAp-Mantecal01, TeAp-N/D1, TeGu-N/D1, TeGu-Terecay01, TeGu-Terecay03 and TeGu-Terecay323. The trypanosomes were expanded in rats and purified by ion exchange chromatography (DEAE-Cellulose) [54]. Parasites were quantified with a hemocitometer and genomic DNA was extracted using a commercial kit (BDtract™, Maxim Biotech, Inc). DNA concentration and purity were determined in a SmartSpect™ 3000 (BioRad). The DNA and PCR amplification products were analyzed by agarose gel electrophoresis under standard conditions.

### Ethical approval

The project was approved by the COBIANIM (IVIC-DIR-1073/12) an advisory body of IVIC with regard to the ethical use of animals in research, in accordance with national and international standards. This committee oversees all research activities at IVIC, requiring the use of animals and wildlife to meet with Venezuelan law and universal ethical values. The Commission assessed the methodological, bioethical and legal aspects of this project by resolution IVIC/N^o^ 1444 [55].

### Microsatellite and Procyclin PE-typing

The PCR amplifications for microsatellite analysis were performed with an Eppendorf Mastercycler, as described [34]. Five microsatellite markers were employed, four that were previously used to characterize trypanosome isolates [34], and a fifth new genetic marker MD2.349-CA amplified with primers MD2.349-CA-F (GCATGCGTGAGGAAGTGAG) and MD2.349-CA-R (GTCCTGTTGGCCGCATTAT) and also a sequence corresponding to the Procyclin PE repeats. The lengths of the PCR products were determined using the Genescan software (Applied Biosystems), and they were sequenced using an ABI 3130 XL (Applied Biosystems) at the C.G.F.B. (Functional Genomic Center of Bordeaux, France).

### Multivariate analysis

Two matrices were generated based on genotyping with microsatellite and Procyclin PE repeats (Additional file 1) and previously published data on *T. brucei brucei* [34]. The categorical values matrix contains all the evaluated microsatellite data, classified from the lowest to the highest number of repeats. The binary values matrix was constructed with the Procyclin PE repeats data, where (0) was the absence and (1) the presence of a determined PE repeat. When each qualitative variable is represented by binary indicator variables for each category, specifying whether an object belongs to it (1) or not (0), multiple correspondence analysis (MCA) can be formulated as a principal component analysis (PCA) of the total set of these indicator variables with respect to some predefined metrics. Thus, just as in PCA, object coordinates can be seen as component scores determined up to a rotation only [56].

By applying this transformation it was possible to use the Hill-Smith [57] method to combine categorical and binary variables to compare all *Trypanosoma* spp. isolates and strains in a Coinertia analysis [58, 59]. This method is a combination of a multiple correspondence analysis (MCA) for categorical data matrix and MCA rotated to principal component analysis (PCA) for binary data matrix. It was implemented with functions of the ADE-4 package from the R software® [60–62].

### PCR amplification of maxicircle genes

Four maxicircle sequences were amplified using either previously reported primers or novel primers designed for this study, based on the complete sequences of the following *T. brucei* genes: Cytochrome b (*cytb*-GenBank Accession N° M17998); Cytochrome oxidase Subunit 1 (*cox1* -GenBank Accession N° M14820); ATP synthase subunit 6 (*a6*-GenBank Accession N° M14820); and NADH Dehydrogenase Subunit 8 (*nd8* -GenBank Accession N° M63820.1). Primer sequences and PCR conditions are shown in Additional file 2: Table S1. The *cytb* and *cox1* gene amplifications were performed with DNA from all nine Venezuelan isolates, while the *a6* and *nd8* amplifications were carried out only with DNA from TeAp-N/D1 and TeGu-N/D1.

The *cytb, cox1, a6* and *nd8* amplicons were purified prior to sequencing using the AccuPrep® PCR Purification Kit (BIONEER®) according to the manufacturer’s instructions. Sequencing of the TeAp-N/D1 genes: *cytb* [GenBank: KP729379), *cox1* [GenBank: KP729381], *a6* [GenBank: KP729385] and *nd8* [GenBank: KP729383] and the corresponding TeGu-N/D1 genes: *cytb* [GenBank: KP729380], *cox1* [GenBank: KP729386], *a6* [GenBank: KP729382] and *nd8* [GenBank: KP729384] was performed by Macrogen (Korea) and Unidad de Estudios Genéticos y Forenses (UEGF-Instituto Venezolano de Investigaciones Científicas, Venezuela).

### Sequence analysis and alignment

Maxicircle sequences from TeAp-N/D1 and TeGu-N/D1 isolates were compared to the corresponding sequences from *T. equiperdum*, *T. brucei* sp*.* and *T. brucei brucei* retrieved from the Genbank database. Sequence analysis was performed using nucleotide BLAST and the sequences were aligned by MUSCLE tools. Phylogenetic trees were constructed using Maximum Parsimony (MP) and Maximum Likelihood (ML) with the MEGA5.1® software [63] using *T. cruzi* as an outgroup. For the ML method, the evolution of the aligned sequences was analyzed.

## Results

We analyzed nine *Trypanosoma* spp. isolates obtained from two Venezuelan states (Apure and Guárico) and from three different hosts: horses, donkeys and capybara. We previously showed that the RAPDs profile of the TeAp-N/D1 and TeGu-N/D1 isolates (horse) were quite distinct from the other seven Venezuelan isolates [52]. To further characterize these strains, all nine *Trypanosoma* spp. isolates were genotyped using microsatellites and Procyclin repeats followed by multivariate analysis, and the two that clustered with *T. equiperdum* (STIB841/OVI), TeAp-N/D1 and TeGu-N/D1, were analyzed for specific maxicircle gene sequences.

### Genotyping

The results of the microsatellite and Procyclin PE repeats analysis of the nine *Trypanosoma* spp. Venezuelan isolates, along with the corresponding data from fifteen *T. evansi*, three *T. equiperdum* and eighteen *T. b. brucei* reference strains are presented in Additional file 1. The Trypanozoon reference strains used in this study originated from Latin America (Colombia and Brazil), Asia (China) and Africa (Chad, Kenya, Ethiopia, Gambia, Ivory Coast, Burkina Faso, Nigeria, Democratic Republic of Congo, Uganda, Tanzania and South Africa).

Five SSR microsatellites and the PE repeats of the PARP genes were used to analyze the nine Venezuelan isolates. The resulting genotypes were compared to reference *T. evansi* and *T. equiperdum* strains, as well as to eighteen previously reported *T. brucei brucei* strains [34], by coinertia analysis.

### Coinertia analysis

**Fig. 1:**
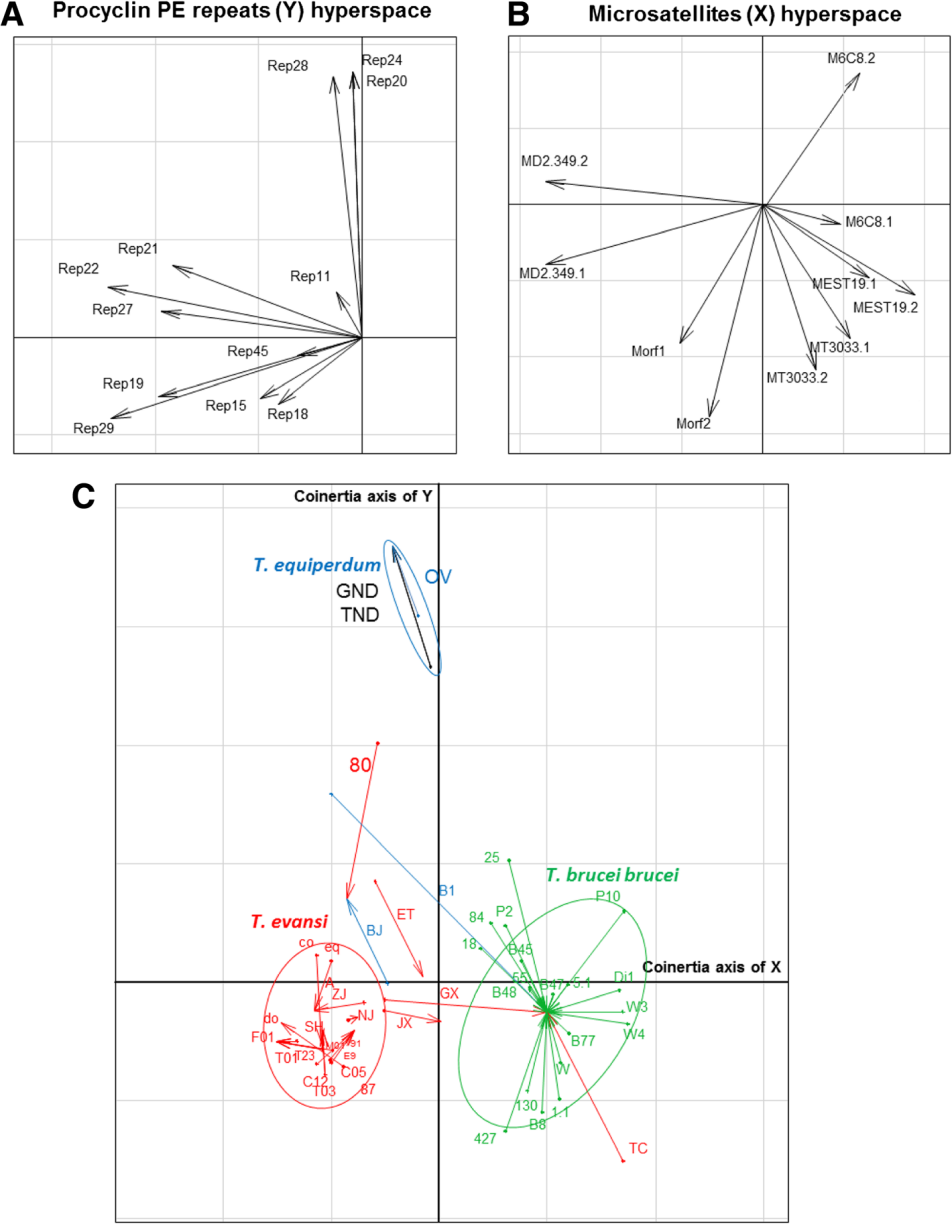
Coinertia analysis by the Hill-Smith method combining microsatellites and Procyclin PE repeats. (**a**) and (**b**) scatterplots represent the coefficients of the combinations of the variables for each data matrix to define the coinertia axes. Separate analyses find axes maximizing inertia in each hyperspace. These axes are projected in the scatterplot (**c**) on which the *Trypanosoma* spp. isolates and reference strains are also projected. The beginning of the arrows is the position of the isolate described by the microsatellite data matrix and the end of the arrow is the position of the isolate described by the procyclin PE repeats. Arrows of the same species were grouped in ellipses of 95 % of variance observed, identifying three groups: *T. evansi* (red), *T. brucei brucei* (green) and *T. equiperdum* (blue). *T. evansi* and *T. equiperdum* isolates that fell outside the major groups were not used to calculate the confidence ellipses. The analysis explained 53.68 % in the microsatellites hyperspace and 22.16 % in the Procyclin PE repeats hyperspace of the observed inertia with a Rv Escoufier similarity coefficient of 0.424415. **C05**: TeAp-Cedral05; **C12**:TeAp-Cedral12; **T03**: TeGu-Terecay03; **F01**: TeAp-ElFrio01; **M01**: TeAp-Mantecal01; **T23**: TeGu-Terecay323; **T01**: TeGu-Terecay01; **TND**: TeAp-N/D1; **GND**: TeGu-N/D1; **E9**: E9/CO; **87**: 2187; **91**: 2191; **A**: A; **do**: dog; **eq**: equi; **co**: coati; **SH**: SH; **ZJ**: ZJ; **NJ**: NJ; **GX**: GX; **JX**: JX; **TC**: TC; **ET**: ET; **80**: KETRI 2480; **OV**: STIB841/OVI; **B1**: BoTat-1.1; **BJ**: BJ; **5.1**: AnTat-5/1; **55**: LM 55; **18**: LM 118; **84**: LM 184; **25**: LM 225; **P10**: KP10; **130**: PTAG 130 (IPR-01130); **P2**: KP2; **Di1**: DiTat-1; **B8**: B8/18; **W3**: SW3/87; **W4**: SW4/87; **W**: SW 161/87; **B45**: STIB 345; **B77**: STIB-777.AE; **1.1**: AnTat-1/1; **427**: EATRO-427; **B47**: STIB247.LFB; **B48**: STIB348

The rotated binary-matrix was constrained with the categorical data matrix by the Hill-Smith method to be able to perform a coinertia analysis that explained 53.68 % and 22.161 % of the observed inertia in the microsatellites hyperspace (X matrix in x axis) and the Procyclin PE repeats hyperspace (Y matrix in y axis), respectively (Fig. 1a and 1b). The two markers, MORF2-CA and MEST19-AT/GT, contributed most to the construction of the coinertia first axis with 80.31 % and 62.58 % on average per allele, respectively. In the second coinertia axis, Repeats 24 and 28 contributed most with 72.67 % and 66.81 %, respectively. Figure 1c revealed three distinct groups, one with *T. evansi* strains, a second group with all the *T. brucei brucei* strains and a third group with the *T. equiperdum* STIB841/OVI strain and the two Venezuelan isolates from this study, TeAp-N/D1 and TeGu-N/D1, shown with black arrows. The remaining seven Venezuelan *Trypanosoma* spp. isolates are close to the *T. evansi* reference strains from the first group. The coinertia analysis of the available genotyping data showed that four of the *T. evansi* strains, namely JX, TC, ET and 80 are genetically distinct. Interestingly, one of them, the TC strain, is closely related to the *T. b. brucei* group (Fig. 1c). The B1 and BJ *T. equiperdum* references strains are different from the STIB841/OVI strain (Fig. 1c). Based on the PE repeats, the *T. evansi* KETRI2480 and *T. equiperdum* BJ strains are closely related (Fig. 1c). The wide distribution of *T. evansi* and *T. equiperdum* strains among distinct groups strongly supports multiple evolutionary origins for these dyskinetoplastic strains.

### Amplification of maxicircle genes

Because the presence of maxicircles is a universally accepted marker to distinguish *T. equiperdum* from *T. evansi,* amplification of two maxicircle genes *(cytb and cox1),* was attempted in the nine Venezuelan Trypanosoma spp. isolates. In agreement with the microsatellite data, no amplification of *cytb* and *cox1* was observed in the seven Venezuelan *Trypanosoma* spp. isolates that belong to the main *T. evansi* cluster: TeAp-Cedral05, TeAp-Cedral12, TeGu-Terecay03, TeAp-El Frío01, TeAp-Mantecal01, TeGu-Terecay323 and TeGu-Terecay01. However both *cytb* and *cox1* could be amplified from DNA of the TeAp-N/D1 and TeGu-N/D1 isolates, as were, two additional maxicircle genes, ATP synthase subunit 6 (*a6)* and NADH dehydrogenase subunit 8 (*nd8).*

### Phylogenetic analysis of four maxicircle genes

Analysis of the sequences of the *cytb, cox1*, *a6* and *nd8* genes amplified from the TeAp-N/D1 and TeGu-N/D1 isolates with BLASTn revealed maximum identities between 98 and 99 % to the orthologous genes from *T. equiperdum* strain STIB842 and BoTat1.1 (Additional file 2: Tables S2-S9). In addition, *cox1* from TeAp-N/D1 was 99 % identical to the corresponding gene from *T. brucei* strain [GenBank: M14820] (Additional file 2: Table S4).

**Fig. 2:**
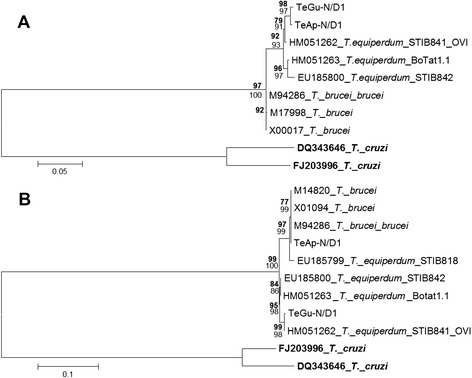
Phylogenetic Relationship of *cytb* and *cox1* gene sequences for two Venezuelan *Trypanosoma* spp. isolates. Phylogenetic trees for the ***cytb*** (**a**) and ***cox1*** (**b**) gene sequences were inferred by Maximum Parsimony (**MP**, bold numbers) and Maximum Likelihood (ML) methods in MEGA 5.1 ®, 500 replicates (bootstrap > 75 % are shown). *T. cruzi* was used as outgroup. The tree topology shown corresponds to the ML method derived from previous evolutionary sequence alignment. *cytb* = Hasegawa-Kishino-Yano (HKY) + Gamma distribution. ***cox1*** = HKY + Invariant sites

**Fig. 3:**
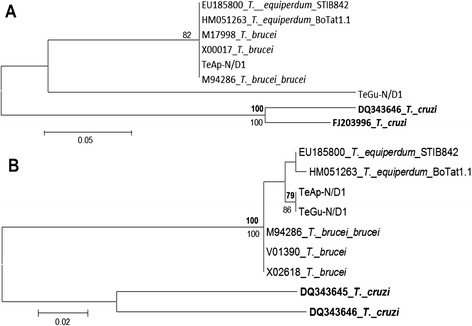
Phylogenetic Relationship of *a6* and *nd8* gene sequences for two Venezuelan *Trypanosoma* spp. isolates. Phylogenetic trees for the ***a6*** (**a**) ***and nd8*** (**b**) **gene sequences** were inferred by Maximum Parsimony (**MP**, bold numbers) and Maximum Likelihood (ML) methods in MEGA 5.1 ®, 500 replicates (bootstrap > 75 % are shown). *T. cruzi* was used as outgroup. The tree topology shown corresponds to the ML method derived from previous evolutionary sequence alignment. ***a6*** = Tamura-Nei. ***nd8*** = Kimura 2-parameter

Phylogenetic trees were constructed using both maximum parsimony and maximum likelihood, with *T. cruzi* as outgroup. The *cytb* analysis showed that TeAp-N/D1 and TeGu-N/D1 are closely related to the *T. equiperdum* STIB841/OVI strain (Fig. 2a). The phylogenetic relationship of *cox1* sequences shows three different subgroups, one including TeAp-N/D1 and the *T. equiperdum* STIB818 and three *T. brucei* strains, a second group comprising TeGu-N/D1 and the *T. equiperdum* STIB841/OVI strain and a third group with the *T. equiperdum* STIB842 and BoTat1.1 strains (Fig. 2b). The phylogenetic construction estimated with the *a6* sequences shows the TeGu-N/D1 strain as a separate group, while TeAp-N/D1 showed identity with three *T. brucei* strains and two *T. equiperdum* STIB842/ BoTat1.1 strains (Fig. 3a). The *nd8* sequences of TeAp-N/D1 and TeGu-N/D1 were identical and related to *T. equiperdum* STIB842 and BoTat1.1 strains (Fig. 3b).

The pattern and multivariate analysis of microsatellite and PE repeats from seven of the *Trypanosoma* spp. isolates, TeAp-Cedral05, TeAp-Cedral12, TeAp-ElFrío01, TeAp-Mantecal01, TeGu-Terecay323, TeGu-Terecay01, TeGu-Terecay03, as well as the lack of amplification of two of the maxicircle genes confirmed that these seven isolates belong to the *T. evansi* group, while the genotype and coinertia analysis of TeAp-N/D1 and TeGu-N/D1 showed that they are closely related to the *T. equiperdum* STIB841/ OVI strain (Table 1). Further, comparison of the sequences of maxicircle gene *cox1* separated the TeAp-N/D1 and TeGu-N/D1 isolates, so that TeAp-N/D1 isolate clustered with *T. equiperdum* STIB818 and *T. brucei brucei*, while the TeGu-N/D1 grouped with the *T. equiperdum* STIB841/OVI.

**Table 1:** Summary of the Venezuelan *T. evansi* and *T. equiperdum* strains characterized in this study

Strain (host)	Microsatellite Genotyping and Coinertia analysis	Maxicircle genes
*T. evansi*		
TeAp-Cedral05 (capybara)	Closely related to *T. evansi* references strains from South America (Brazil and Colombia) and Asia (China).	n.a.
TeAp-Cedral12 (capybara)		n.a.
TeAp-ElFrío01 (capybara)		n.a.
TeAp-Mantecal01 (horse)		n.a.
TeGu-Terecay03 (donkey)		n.a.
TeGu-Terecay01 (donkey)		n.a.
TeGu-Terecay323 (donkey)		n.a.
*T. equiperdum*		
TeAp-N/D1 (horse)	Closely related to the *T. equiperdum* STIB841/ OVI strain	Positive for *cytb, cox1, a6 and nd8*
TeGu-N/D1 (horse)	Closely related to the *T. equiperdum* STIB841/OVI strain.	Positive for *cytb, cox1, a6 and nd8*

na: not amplified by PCR. The nomenclature, natural hosts and molecular characteristics of the nine Venezuelan strains included in this study are presented. The geographical origin of the strains has been previously reported [52]

## Discussion

The classification of trypanosomes within the Trypanozoon sub-genera was originally based on morphological and morphometrical criteria, as well as clinical manifestations, host range and geographical distribution. In the last several years, molecular markers such as microsatellite loci and sequence analysis of the rRNA and gGAPDH genes have been used to describe the evolutionary relationships among organisms. This has led to the re-evaluation of what constitutes a trypanosome species [5, 34, 64, 65]. The lack of maxicircle genes in *T. evansi* has been used to differentiate it from *T. equiperdum* [33], both being considered as petite mutants of *T. brucei* [51]. Subtle genomic changes were found between the akinetoplastic strain *T. evansi* STIB805 and the *T. b. brucei* TREU 927/4 strain, supporting the view that *T. evansi* should be considered a subspecies of *T. brucei* [66]. The species or subspecies status of both *T. evansi* and *T. equiperdum* has been debated by several authors and continues to be polemical [4, 16, 51, 66, 67].

Using RAPD analysis, we previously showed that nine Venezuelan *Trypanosoma* spp. isolates cluster into two separate groups, one with seven isolates that share a similar genetic pattern and a second, distinct group of two horse isolates, TeAp-N/D1 and TeGu-N/D1 that appeared identical except with one of the forty random primers [52]. Since these nine Venezuelan *Trypanosoma* spp. isolates were presumed to belong to the Trypanozoon group, we used microsatellites markers and PARP-PE repeats to compare them to *T. evansi* and *T. equiperdum* reference strains. These loci exhibited limited polymorphism among seven of our isolates and the *T. evansi* reference strains from various hosts, horse, dog, coati, bovine, buffalo, mule and camels and geographical origins, South America, Asia and Africa. However, the microsatellite analysis clearly clustered two of the Venezuelan *Trypanosoma* spp. horse isolates with the *T. equiperdum* STIB841/OVI strain from South Africa.

Coinertia analysis of the microsatellite and PE repeats revealed that seven of the nine Venezuelan isolates closely match eleven *T. evansi* reference strains from around the world, while TeAp-N/D1 and TeGu-N/D1 are identical to the South African *T. equiperdum* STIB841/OVI strain. Principal Component Analysis (PCA) of microsatellite markers showed that this *T. equiperdum* strain is closely related to the *T. brucei brucei* Kiboko group [66]. In agreement with previous studies of Trypanozoon microsatellite loci [34], our coinertia analysis showed that BoTat-1.1 and BJ were highly heterogeneous *T. equiperdum* strains, genetically distant from the group that included the two Venezuelan *T. equiperdum* strains and STIB841/OVI. Four independent *T. evansi/T. equiperdum* genotypes have been recently described by Carnes *et al* [66]. They classified two of the reference *T. equiperdum* strains included in this study, BoTat-1.1 (Teq21) and STIB841/OVI, within groups 2 and 3, respectively.

Since *T. equiperdum* is distinguished from *T. evansi* by the presence of partially deleted maxicircles [14, 51, 67], we amplified and sequenced maxicircle genes from the TeAp-N/D1 and TeGu-N/D1 isolates and analyzed the resulting phylogenetic relationships. In agreement with previous reports, the two Venezuelan *T. equiperdum* strains analyzed in this study, TeAp-N/D1 and TeGu-N/D1, have retained at least four maxicircle genes [51]. ND8 and A6 constitute complexes I and V of the oxidative phosphorylation system, and their expression is essential in the *T. brucei* bloodstream form [68, 69], as is the expression of *cytb* [70]. A6 is important for maintaining the mitochondrial membrane potential and several mutations that affect its function have been described in diskinetoplastic trypanosomes [71–73]. Mitochondrial genes have been proposed as excellent molecular markers for discriminating closely related species [74–76]. The four maxicircle genes from the TeAp-N/D1 and TeGu-N/D1 isolates revealed a close relationship to both *T. brucei* and *T. equiperdum* strains. The phylogenetic analysis of *cox1* gene sequences is concordant with the four distinct groups of *T. evansi* and *T. equiperdum* strains that suggest four independent origins of these diskinetoplastic parasites [66]. Interestingly, while the microsatellite loci and Procyclin PE repeats and coinertia analyses showed identical genotypes for TeAp-N/D1 and TeGu-N/D1, the *cox1* and *a6* mitochondrial markers separated them into different clusters. These results confirm the first molecular report of *T. equiperdum* strains (TeAp-N/D1 and TeGu-N/D1) isolated in Venezuela or in any part of Latin America.

Interestingly, *T. equiperdum* is not a typical American parasite but dourine, a sexually transmitted chronic disease in horses, mules and donkeys [5, 17], has been sporadically reported in the American continent from Canada (1921) to the USA (1934) and Mexico (1973) [77]. In Venezuela, clinical dourine was first recorded by the presence of dourine plaques in a domestic male horse, but no parasites were observed [78]. The actual geographical distribution of dourine is not known and trade restrictions appear to limit notification of the disease. The disease was widespread in the past but was eradicated from many countries of Europe after the 1940s [22, 79]. Currently, the disease is endemic in parts of Africa, Asia and Russia, and dourine outbreaks or incidents have been occasionally reported in the Middle East and Europe [16, 80–82]. However, dourine may exist in areas where diagnostic tests are not routinely performed [3, 17]. In 2011, seven dourine outbreaks were confirmed in various regions of Italy [18–21, 83], all linked to the movement of breeding animals showing the characteristic plaques and lesions. The presence of *T. equiperdum* was confirmed by RT-PCR.

*T. equiperdum* is morphologically indistinguishable from *T. evansi* and *T. brucei*, [5, 17]. *T. evansi* was initially proposed to have evolved from an ancestral *T. brucei* when infected camels were introduced to Glossina-free areas [5, 14] and is characterized by the absence of maxicircle structures [45, 84]. Several years ago, Claes *et al* [16] proposed that *T. equiperdum* does not exist as a separate species and that extant strains are either *T.b. equiperdum,* or misidentified *T.b. brucei* or *T. evansi* strains. Based on genetic analysis, other authors have also proposed that *T. evansi* and *T. equiperdum* should be considered sub-species of *T. brucei* [16, 17, 51, 65, 66]. In contrast, Desquesnes et al [4] recommend keeping the current species status for *T. evansi* and *T. equiperdum*, in agreement with the rules of the international code for zoological nomenclature and based on their significant biological differences. Further genomic analysis of the two Venezuelan *T. equiperdum* strains should shed new light on the evolution, origin and pathogenic effects of these trypanosomes.

The evolution of *T. evansi* and *T. equiperdum* has been revisited in recent years. Lun *et al* [67] proposed two sequential steps in the spreading of *T. brucei* out of Africa, the first involved the homogenization of minicircles in the *T. b. brucei* bloodstream form and the loss of the ability to differentiate within the insect vector, resulting in *T. b. equiperdum*. Over generations, the gradual loss of maxicircles occurred due to the lack of selective pressure to preserve them, giving rise to *T. b. equiperdum* and eventually *T. b. evansi,* which lacks all maxicircles. An alternative model proposed that an ancestral trypanosome lost maxicircle genes in three [85] or four independent occasions [66], to generate stable diskinetoplastic forms, a loss that is compensated by distinct mutations on the ATP synthase γ-subunit [85]. A third, alternative hypothesis proposes that *T. b. evansi* and *T. b. equiperdum* underwent separate evolutionary processes from a *T. b. brucei* ancestor [17]. Our results are quite consistent with the existence of partially distinct evolutionary lineages, with the two new Venezuelan *T. equiperdum* strains, TeAp-N/D1 and TeGu-N/D1 corresponding to the recently proposed *T. equiperdum* groups 1 and 3, respectively [66].

## Conclusion

**Fig. 4:**
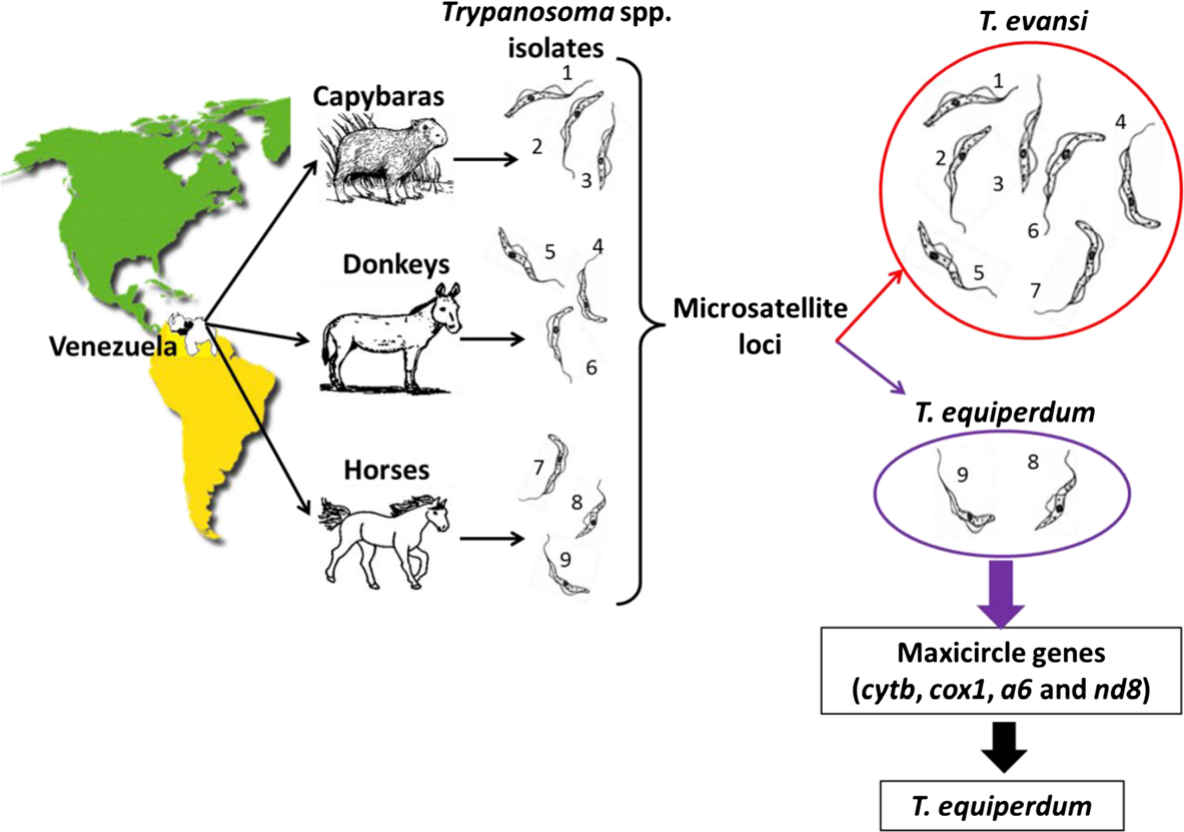
Microsatellites and four maxicircle genes were used to characterize nine *Trypanosoma* spp. Venezuelan isolates. Seven isolates were shown to be closely related to *T. evansi* reference strains, while two were classified as *T. equiperdum*

The microsatellite data divided the nine Venezuelan trypanosoma isolates into two groups, one closely related to *T. evansi* and a second, closely related to *T. equiperdum* (STIB841/OVI). The classification of TeAp-N/D1 and TeGu-N/D1 as *T. equiperdum* is supported by the sequences of their four maxicircle genes that are nearly identical to the orthologous *T. brucei* and *T. equiperdum* genes. Coinertia analysis of microsatellites and Procyclin PE repeats also place these two Venezuelan isolates close to the STIB841/OVI *T. equiperdum* strain. Phylogenetic analysis of the sequences of the *cox1* gene, an exceptional discriminative molecular marker, separated the two isolates: TeAp-N/D1 clustered with the *T. equiperdum* STIB818 strain; while TeGu-N/D1 was grouped with the *T. equiperdum* STIB841/OVI strain. This constitutes the first molecular report of *T. equiperdum* strains isolated in Latin America (Fig. 4).

## Additional files

Additional file 1:
**Table S2-S9.** Microsatellites from Venezuelan *Trypanosoma* spp. isolates,*T. evansi,* *T. equiperdum*and*T. brucei brucei*strains. The microsatellite loci MORF2-CA, M6C8-CA, MT3033-AC/TC, MEST19-AT/GT, MD2.349-CA and PE repeats of the PARP genes of *T. evansi* strains from South America (Colombia and Brazil), Asia (China) and Africa (Chad, Ethiopia and Kenya), *T. equiperdum* strains from Africa (South Africa) and Asia (China) and *T. b. brucei* from (Kenya, Gambia, Ivory Coast, Burkina Faso, Nigeria, Democratic Republic of Congo, Uganda and Tanzania). Further information on the strains can be found in the following references: *T. evansi* strains: E9/CO in [86], 2187 and 2191 in [87]; A, dog, equi Asia (China) and Africa (South Africa and Morocco), as well as nine Venezuelan *Trypanosoma* spp. isolates are presented. nd: not determined. and coati (DNA provided by Alberto M.R. Dávila, unpublished results), SH, ZJ, NJ, GX, JX, TC, ET and in [87]; KETRI2480 in [88, 89]. *T. equiperdum* strains: OVI in [87, 90]; BoTat-1.1 in [90, 91]; BJ in [48, 87]. (XLSX 41 kb) Additional file 2:
**Table S1.** Primers and PCR conditions used in this study. **Figure S1**. Diagram of amplicons and primers used to amplify the four maxicircle genes used in this study. **Table S2.** BLASTn analyses of cytochrome b gene sequences from the TeAp-N/D1 isolate [GenBank Accession N° KP729379]. 1056 bp. **Table S3.** BLASTn analyses of cytochrome b gene sequences from the TeGu-N/D1 isolate [GenBank Accession N° KP729380].1056 bp. **Table S4.** BLASTn analyses of Cytochrome Oxidase Subunit 1 gene sequences from the TeAp-N/D1 isolate [GenBank Accession N° KP729381]. 1647 bp. **Table S5.** BLASTn analyses of Cytochrome Oxidase Subunit 1 gene sequences from the TeGu-N/D1 isolate sequence [GenBank Accession N° KP729386]. 1530 bp. **Table S6.** BLASTn analyses of ATP synthase subunit 6 gene sequences from the TeAp-N/D1 isolate sequence [GenBank Accession N° KP729385]. 285 bp. **Table S7.** BLASTn analyses of ATP synthase subunit 6 gene sequences from the TeGu-N/D1 isolate sequence [GenBank Accession N° KP729382]. 285 bp. **Table S8.** BLASTn analyses of NADH dehydrogenase subunit 8 gene sequences from the TeAp-N/D1 isolate sequence [GenBank Accession N° KP729383]. 342 bp. **Table S9.** BLASTn analyses of NADH dehydrogenase subunit 8 gene sequences from the TeGu-N/D1 isolate sequence [GenBank: KP729384]. 348 bp. (DOCX 69 kb)
