# Erratum to: Molecular characterization and classification of *Trypanosoma* spp. Venezuelan isolates based on microsatellite markers and kinetoplast maxicircle genes

**DOI:** 10.1186/s13071-015-1177-7

**Published:** 2015-10-29

**Authors:** E. Sánchez, T. Perrone, G. Recchimuzzi, I. Cardozo, N. Biteau, P. M. Aso, A. Mijares, T. Baltz, D. Berthier, L. Balzano-Nogueira, M. I. Gonzatti

**Affiliations:** Laboratorio de Fisiología de Parásitos. Centro de Biofísica y Bioquímica, Instituto Venezolano de Investigaciones Científicas, Caracas, Venezuela; Grupo de Bioquímica e Inmunología de Hemoparásitos. Departamento de Biología Celular, Universidad Simón Bolívar, Caracas, 1080 Venezuela; Laboratoire de Microbiologie Fondamentale et Pathogénicité, Université Bordeaux. UMR-CNRS 5234, 146, Rue Léo Saignat, Bordeaux, 33076, Cedex France; CIRAD, UMR InterTryp, Montpellier, F-34398 France; Laboratorio de Biometría y Estadística, Área de Agricultura y Soberanía Alimentaria, Instituto de Estudios Avanzados, Caracas, 1015A Venezuela

Unfortunately, the original version of this article [1] contained an error. Figure 1 in the original article, corresponded to the first coinertia analysis that was carried out with no data on the procyclin PE repeats for the *T. brucei brucei* strains. After including these data, the coinertia analysis was modified both in the directionality of the arrows in the Y Hyperspace and in the biplot generated by the interaction of the two coinertia axes. The modified coinertia analysis is included in Fig. 1.

**Fig. 1:**
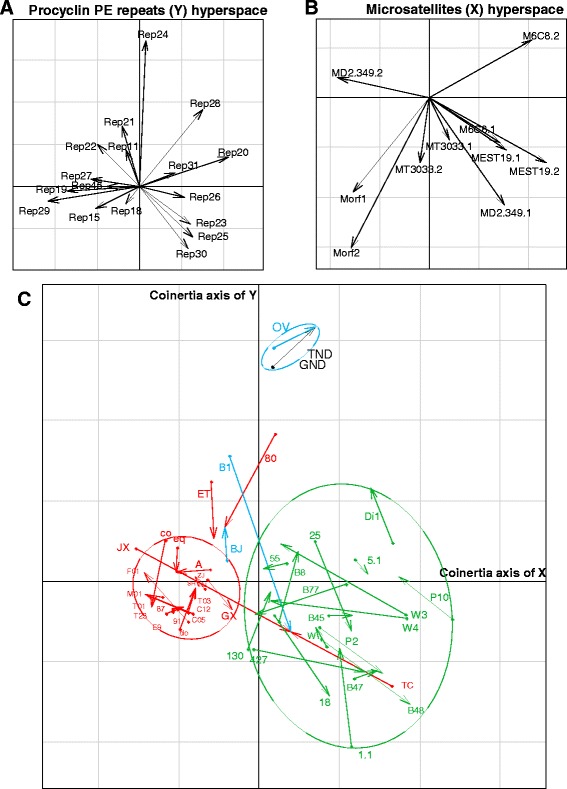
Coinertia analysis by the Hill-Smith method combining microsatellites and Procyclin PE repeats. **a** and **b** scatterplots represent the coefficients of the combinations of the variables for each data matrix to define the coinertia axes. Separate analyses find axes maximizing inertia in each hyperspace. These axes are projected in the scatterplot (**c**) on which the *Trypanosoma* spp. isolates and reference strains are also projected. The beginning of the arrows is the position of the isolate described by the microsatellite data matrix and the end of the arrow is the position of the isolate described by the procyclin PE repeats. Arrows of the same species were grouped in ellipses of 95 % of variance observed, identifying three groups: *T. evansi* (red), *T. brucei brucei* (green) and *T. equiperdum* (blue). *T. evansi* and *T. equiperdum* isolates that fell outside the major groups were not used to calculate the confidence ellipses. The analysis explained 53.68 % in the microsatellites hyperspace and 22.16 % in the Procyclin PE repeats hyperspace of the observed inertia with a Rv Escoufier similarity coefficient of 0.424415. **C05**: TeAp-Cedral05; **C12**:TeAp-Cedral12; **T03**: TeGu-Terecay03; **F01**: TeAp-ElFrio01; **M01**: TeAp-Mantecal01; **T23**: TeGu-Terecay323; **T01**: TeGu-Terecay01; **TND**: TeAp-N/D1; **GND**: TeGu-N/D1; **E9**: E9/CO; **87**: 2187; **91**: 2191; **A**: A; **do**: dog; **eq**: equi; **co**: coati; **SH**: SH; **ZJ**: ZJ; **NJ**: NJ; **GX**: GX; **JX**: JX; **TC**: TC; **ET**: ET; **80**: KETRI 2480; **OV**: STIB841/OVI; **B1**: BoTat-1.1; **BJ**: BJ; **5.1**: AnTat-5/1; **55**: LM 55; **18**: LM 118; **84**: LM 184; **25**: LM 225; **P10**: KP10; **130**: PTAG 130 (IPR-01130); **P2**: KP2; **Di1**: DiTat-1; **B8**: B8/18; **W3**: SW3/87; **W4**: SW4/87; **W**: SW 161/87; **B45**: STIB 345; **B77**: STIB-777.AE; **1.1**: AnTat-1/1; **427**: EATRO-427; **B47**: STIB247.LFB; **B48**: STIB348

## References

[1] Sánchez E, Perrone T, Recchimuzzi G, Cardozo I, Biteau N, Aso PM (2015). Molecular characterization and classification of *Trypanosoma* spp. Venezuelan isolates based on microsatellite markers and kinetoplast maxicircle genes. Parasites and Vectors.

