# Supplementary material for: Molecular characterization and classification of Trypanosoma spp. Venezuelan isolates based on microsatellite markers and kinetoplast maxicircle genes
Source: Parasit Vectors. 2015 Oct 15;8:536. doi: 10.1186/s13071-015-1129-2 (PMC4607141; doi:10.1186/s13071-015-1129-2)
Supplement: Additional file 2: — Table S1. Primers and PCR conditions used in this study. Figure S1. Diagram of amplicons and primers used to amplify the four maxicircle genes used in this study. Table S2. BLASTn analyses of cytochrome b gene sequences from the TeAp-N/D1 isolate [GenBank Accession N° KP729379]. 1056 bp. Table S3. BLASTn analyses of cytochrome b gene sequences from the TeGu-N/D1 isolate [GenBank Accession N° KP729380].1056 bp. Table S4. BLASTn analyses of Cytochrome Oxidase Subunit 1 gene sequences from the TeAp-N/D1 isolate [GenBank Accession N° KP729381]. 1647 bp. Table S5. BLASTn analyses of Cytochrome Oxidase Subunit 1 gene sequences from the TeGu-N/D1 isolate sequence [GenBank Accession N° KP729386]. 1530 bp. Table S6. BLASTn analyses of ATP synthase subunit 6 gene sequences from the TeAp-N/D1 isolate sequence [GenBank Accession N° KP729385]. 285 bp. Table S7. BLASTn analyses of ATP synthase subunit 6 gene sequences from the TeGu-N/D1 isolate sequence [GenBank Accession N° KP729382]. 285 bp. Table S8. BLASTn analyses of NADH dehydrogenase subunit 8 gene sequences from the TeAp-N/D1 isolate sequence [GenBank Accession N° KP729383]. 342 bp. Table S9. BLASTn analyses of NADH dehydrogenase subunit 8 gene sequences from the TeGu-N/D1 isolate sequence [GenBank: KP729384]. 348 bp. (DOCX 69 kb) [file 13071_2015_1129_MOESM2_ESM.docx]

**Additional table A1. Primers and PCR conditions used in this study.**

| **Name** | **Sequence 5’- 3’** | **Annealing T (°C)** | **PCR Mix Nº** | **PCR protocol Nº** | **Reference** |
| --- | --- | --- | --- | --- | --- |
| **Cytochrome b** | | | | | |
| MaxiCyt1 | AGCGGAGAAAAAAGAAAGGGT | 52 | 1 | 1 | Perrone, 2003 |
| MaxiCyt2 | CTAATCTAACCTACACACTATC |  |  |  |  |
| MaxiCyt1 | AGCGGAGAAAAAAGAAAGGGT | 52 | 1 | Nº 1 for TeGu-N/D1. Nº 2 for TeAp-N/D1 | Perrone, 2003 |
| MaxiCyt1.1 | TCACAAAATGCATCAGAACTCA |  |  |  | Designed in this study |
| MaxiCyt0.2 | ACATGTGTTATTACCATTTA | 52 | 1 | 1 | Designed in this study |
| MaxiCyt2 | CTAATCTAACCTACACACTATC |  |  |  | Perrone, 2003 |
| **Cytochrome Oxidase Subunit 1** | | | | | |
| COX0.1 | CAGTTTTGTTTAACACAGTTATTATCA | 52 | 1 | Nº 1 for TeGu-N/D1. Nº 2 for TeAp-N/D1 | Designed in this study |
| COX 0.2 | GCTGGTGGATTAACTGGC |  |  |  |  |
| COX 1 | CCCTACAACCAGCACCAAGT | 52 | Nº 1 with exception of 0,2 µM primers | 1 | Njiru et al, 2006 |
| COX 2 | TTCACATGGGTTGATTATGG |  |  |  |  |
| COX 1.1 | GGAAATACCATATCGGGAAAC | 52 | Nº 2 for TeAp-N/D1. There was no amplification in TeGu-N/D1 | Nº 1 for TeAp-N/D1.  There was no amplification in TeGu-N/D1 | Designed in this study |
| COX 2.1 | GGGTTGATGGAATTTTGG |  |  |  |  |
| **ATP Synthase Subunit 6** | | | | | |
| A6F | AGGAATTTTGGGCGGAAGA | 56 | 1 | Nº 1 except for annealing temperature= 56°C | Lai et al, 2008 |
| A6R | CCCTAACCTTTCCTGCTC |  |  |  |  |
| **NADH Dehydrogenase Subunit 8** | | | | | |
| TbCR1.3 | GTGCCACTTTAATTTAAAACTGCTTAAGCC | 50 | Nº 1 with excepción of 0,2 µM primers | Nº 3 | Souza et al, 1992 |
| TbCR1.8 | GTCGACATCGATTCAATTTAATAATTTTAAGTTTTGG |  |  |  |  |

**PCR conditions used in this study:**

PCR Mix Nº 1: 1X PCR buffer, 0.05U/µl Taq polymerase and 1.5mM MgCl_2_ (Invitrogen), 0.2mM dNTPs (Promega) and 0.8µM primers.

PCR Mix Nº 2: 1X PCR buffer, 0.1U/µl Taq polymerase and 4mM MgCl_2_, 0.2mM dNTPs (Promega) and 0.8µM primers.

PCR Protocol Nº 1: 95°C x 5min (1), 95°C x 45sec (2), 52°C x 45sec (3), 72°C x 45sec (4), 40 cycles from step (2) to step (4), 72°C x 5min (5).

PCR Protocol Nº 2: 95°C x 5min (1), 95°C x 1min (2), 52°C x 2min (2), 72°C x 2min (2), 40 cycles step (2) to step (4), 72°C x 10min (5).

PCR Protocol Nº 3: 95°C x 5min (1), 95°C x 1min (2), 50°C x 30sec (3), 72°C x 30sec (4), 35 cycles from (2) to (4), 72°C x 10min (5).

All the PCR were performed in a Mastercycler® (Eppendorf).

**
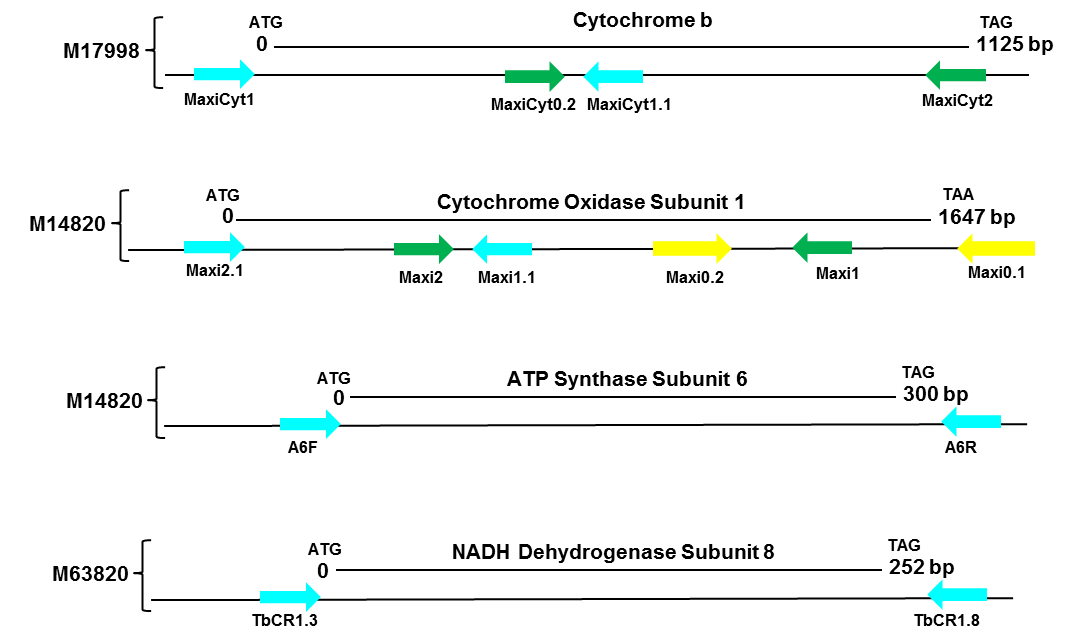
**

**Additional figure A1. Diagram of amplicons and primers used to amplify the four maxicircle genes used in this study.** *Trypanosoma brucei* GenBank Accession N° M17998, M14820 and M63820: Unedited Cytochrome b gene (*cytb*) (599 bp, MaxiCyt1-MaxiCyt1.1; 571 bp, MaxiCyt0.2-MaxiCyt2; 1079 bp, MaxiCyt1-MaxiCyt2), unedited Cytochrome Oxidase Subunit 1 gene (*cox1*) 358 bp, Maxi2.1-Maxi1.1; 571 bp, Maxi2-Maxi1; 626 bp, Maxi0.2-Maxi0.1). Pre-edited ATP Synthase Subunit 6 and NADH Dehydrogenase Subunit 8 genes (*a6*, *nd8*). bp = base pairs.

**Additional table A2. BLASTn analyses of cytochrome b gene sequences from the TeAp-N/D1 isolate [GenBank Accession Nº KP729379].** 1056 bp.

| **GenBank^®^  Nº (strain)** | **Species** | **Reference** | **Identities** | **Score (Bits)** | **E Value** | **% Identity** |
| --- | --- | --- | --- | --- | --- | --- |
| HM051263 (BoTat1.1) | *T. equiperdum* | Lai and Lun, 2010 (unpublished) | 1030/1047 | 1842 (997) | 0,0 | 98 |
| EU185800 (STIB842) | *T. equiperdum* | Lai et al, 2008 | 1025/ 1047 | 1814 (982) | 0,0 | 98 |
| M94286 | *T. brucei brucei* | Feagin et al, 1985 | 1014/1047 | 1753 (949) | 0,0 | 97 |
| X00017 | *T. brucei* | Benne et al, 1983 | 1014/1047 | 1753 (949) | 0.0 | 97 |
| M17998 | *T. brucei* | Johnson et al, 1984 | 1013/ 1047 | 1748 (946) | 0.0 | 97 |
| HM051262 (STIB841/OVI) | *T. equiperdum* | Lai and Lun, 2010  (unpublished) | 680/ 688 | 1225 (663) | 0.0 | 99 |

**Additional table A3. BLASTn analyses of cytochrome b gene sequences from the TeGu-N/D1 isolate [GenBank Accession Nº KP729380].**1056 bp.

| **GenBank^®^ Nº (strain)** | **Species** | **Reference** | **Identities** | **Score (Bits)** | **E Value** | **% Identity** |
| --- | --- | --- | --- | --- | --- | --- |
| HM051263 (BoTat1.1) | *T. equiperdum* | Lai and Lun, 2010 (unpublished) | 1035/1049 | 1857 (1005) | 0.0 | 99 |
| EU185800 (STIB842) | *T. equiperdum* | Lai et al, 2008 | 1030/ 1049 | 1829 (990) | 0.0 | 98 |
| M94286 | *T. brucei brucei* | Feagin et al, 1985 | 1019/1049 | 1768 (957) | 0.0 | 97 |
| X00017 | *T. brucei* | Benne et al, 1983 | 1019/ 1049 | 1768 (957) | 0.0 | 97 |
| M17998 | *T. brucei* | Johnson et al, 1984 | 1018/ 1049 | 1762 (954) | 0.0 | 97 |
| HM051262 (STIB841/OVI | *T. equiperdum* | Lai and Lun, 2010  (unpublished) | 678/ 688 | 1212 (656) | 0.0 | 99 |

**Additional table A4. BLASTn analyses of Cytochrome Oxidase Subunit 1 gene sequences from the TeAp-N/D1 isolate [GenBank Accession Nº KP729381].** 1647 bp.

| **GenBank^®^ Nº (strain)** | **Species** | **Reference** | **Identities** | **Score (Bits)** | **E**  **Value** | **% Identity** |
| --- | --- | --- | --- | --- | --- | --- |
| M14820 | *T. brucei* | Payne et al, 1985 | 1626/ 1643 | 2942 (1593) | 0.0 | 99 |
| X01094 | *T. brucei* | Hensgens et al, 1984 | 1626/ 1643 | 2942 (1593) | 0.0 | 99 |
| M94286 | *T. brucei brucei* | Hensgens et al, 1984 | 1623/1643 | 2924 (1583) | 0.0 | 99 |
| EU185800 (STIB842) | *T. equiperdum* | Lai et al, 2008 | 1604/ 1643 | 2820 (1527) | 0.0 | 98 |
| HM051263 (BoTat1.1) | *T. equiperdum* | Lai and Lun, 2010  (unpublished) | 1267/1296 | 2235 (1210) | 0.0 | 98 |
| HM051262 (STIB842/OVI) | *T. equiperdum* | Lai and Lun, 2010  (unpublished) | 1264/ 1297 | 221 (1199) | 0.0 | 97 |
| EU185799 (STIB818) | *T. equiperdum* | Lai et al, 2008 | 1242/1270 | 2187 (1184) | 0.0 | 98 |

**Additional table A5. . BLASTn analyses of Cytochrome Oxidase Subunit 1 gene sequences from the TeGu-N/D1 isolate sequence [GenBank Accession Nº KP729386].** 1530 bp.

| **GenBank^®^  Nº (strain)** | **Species** | **Reference** | **Identities** | **Score (Bits)** | **E Value** | **% Identity** |
| --- | --- | --- | --- | --- | --- | --- |
| EU185800 (STIB842) | *T. equiperdum* | Lai et al, 2008 | 1331/ 1345 | 2407 (1303) | 0.0 | 99 |
| HM051262 (STIB842/OVI) | *T. equiperdum* | Lai and Lun, 2010  (unpublished) | 1299/ 1302 | 2388 (1293) | 0.0 | 99 |
| HM051263 (BoTat1.1) | *T. equiperdum* | Lai and Lun, 2010  (unpublished) | 1289/1302 | 2333 (1263) | 0.0 | 99 |
| M14820 | *T. brucei* | Payne et al, 1985 | 1307/ 1345 | 2274 (1231) | 0.0 | 97 |
| X01094 | *T. brucei* | Hensgens et al, 1984 | 1307/ 1345 | 2274 (1231) | 0.0 | 97 |
| M94286 | *T. brucei brucei* | Hensgens et al, 1984 | 1304/1345 | 2254 (1220) | 0.0 | 97 |
| EU185799 (STIB818) | *T. equiperdum* | Lai et al, 2008 | 932/965 | 1596 (864) | 0.0 | 97 |

**Additional table A6. BLASTn analyses of ATP synthase subunit 6 gene sequences from the TeAp-N/D1 isolate sequence [GenBank Accession Nº KP729385].** 285 bp.

| **GenBank^®^  Nº (strain)** | **Species** | **Reference** | **Identities** | **Score (Bits)** | **Value E** | **% Identity** |
| --- | --- | --- | --- | --- | --- | --- |
| EU185800 (STIB842) | *T. equiperdum* | Lai et al, 2008 | 278/ 282 | 278 (282) | 2e-139 | 99 |
| M94286 | *T. brucei brucei* | Bhat et al, 1990 | 275/ 283 | 481 (260) | 3e-132 | 97 |
| M17998 | *T. brucei* |  | 275/ 283 | 481 (260) | 3e-132 | 97 |
| X00017 | *T. brucei* | Benne et al, 1983 | 275/ 283 | 481 (260) | 3e-132 | 97 |
| HM051263 (BoTat1.1) | *T. equiperdum* | Lai and Lun, 2010  (unpublished) | 54/ 54 | 100 (54) | 1e-17 | 100 |

**Additional table A7. BLASTn analyses of ATP synthase subunit 6 gene sequences from the TeGu-N/D1 isolate sequence [GenBank Accession Nº KP729382].** 285 bp.

| **GenBank^®^ Nº** | **Species** | **Reference** | **Identities** | **Score (Bits)** | **E Value** | **% Identity** |
| --- | --- | --- | --- | --- | --- | --- |
| EU185800 (STIB842) | *T. equiperdum* | Lai et al, 2008 | 240/ 244 | 427 (231) | 4e-116 | 98 |
| M94286 | *T. brucei brucei* | Bhat et al, 1990 | 239/ 245 | 416 (225) | 9e-113 | 98 |
| M17998 | *T. brucei* | ND | 239/ 245 | 416 (225) | 9e-113 | 98 |
| X00017 | *T. brucei* | Benne et al, 1983 | 239/ 245 | 416 (225) | 9e-113 | 98 |

**Additional table A8. BLASTn analyses of NADH dehydrogenase subunit 8 gene sequences from the TeAp-N/D1 isolate sequence [GenBank Accession Nº KP729383].** 342 bp.

| **GenBank^®^  Nº (strain)** | **Species** | **Reference** | **Identities** | **Score (Bits)** | **E Value** | **% Identity** |
| --- | --- | --- | --- | --- | --- | --- |
| EU185800 (STIB842) | *T. equiperdum* | Lai et al, 2008 | 311/ 314 | 562 (304) | 1e-156 | 99 |
| V01390 | *T. brucei* | Eperon et al, 1983 | 309/ 313 | 555 (300) | 2e-154 | 99 |
| M94286 | *T. brucei brucei* | Bhat et al, 1990 | 309/ 313 | 555 (300) | 2e-154 | 99 |
| X02618 (EATRO164) | *T. brucei* | Feagin et al, 1985 | 309/ 313 | 555 (300) | 2e-154 | 99 |
| HM051263 (BoTat1.1) | *T. equiperdum* | Lai and Lun, 2010  (unpublished) | 241/ 243 | 436 (236) | 8e-119 | 99 |

**Additional table A9. BLASTn analyses of NADH dehydrogenase subunit 8 gene sequences from the TeGu-N/D1 isolate sequence [GenBank: KP729384].** 348 bp.

| **GenBank^®^  Nº (strain)** | **Species** | **Reference** | **Identities** | **Score (Bits)** | **E Value** | **% Identity** |
| --- | --- | --- | --- | --- | --- | --- |
| EU185800 (STIB842) | *T. equiperdum* | Lai et al, 2008 | 300/ 305 | 532 (288) | 1e-147 | 98 |
| V01390 | *T. brucei* | Eperon et al, 1983 | 299/ 304 | 531 (287) | 4e-147 | 98 |
| M94286 | *T. brucei brucei* | Bhat et al, 1990 | 299/ 304 | 531 (287) | 4e-147 | 98 |
| X02618 (EATRO164) | *T. brucei* | Feagin et al, 1985 | 299/ 304 | 531 (287) | 4e-147 | 98 |
| HM051263 (BoTat1.1) | *T. equiperdum* | Lai and Lun, 2010  (unpublished) | 241/ 243 | 436 (236) | 9e-119 | 99 |
